# Hard Water, More Elastic Arteries: A Case Study from Krupina District, Slovakia

**DOI:** 10.3390/ijerph16091521

**Published:** 2019-04-29

**Authors:** Stanislav Rapant, Veronika Cvečková, Katarína Fajčíková, Igor Hajdúk, Edgar Hiller, Beáta Stehlíková

**Affiliations:** 1 Department of Geochemistry, Faculty of Natural Sciences, Comenius University in Bratislava, Ilkovičova 6, 842 15 Bratislava, Slovakia; veronika.cveckova@uniba.sk (V.C.); edgar.hiller@uniba.sk (E.H.); 2 Magistrate of the Capital City of Bratislava, Primaciálne nám. 1, 814 99 Bratislava, Slovakia; katarina.fajcikova@bratislava.sk; 3 Institute for Work Rehabilitation of Disabled People, Mokrohájska 1, 842 40 Bratislava, Slovakia; igor.hajduk@iprba.sk; 4 Faculty of Economics of Business, Pan-European University, Tematínska 10, 851 05, Bratislava 5, Slovakia; stehlikovab2@gmail.com

**Keywords:** cardiovascular diseases, arterial stiffness, human health, drinking water, calcium, magnesium, water hardness

## Abstract

The protective role of hard drinking water against cardiovascular diseases is well documented by numerous studies. This article describes the impact of Ca and Mg contents in the drinking water with different water hardness on the cardiovascular system (arterial stiffness, arterial age) of residents of the Krupina district, the Slovak Republic. The research was based on the measurements of arterial stiffness, including the measurements of aortic pulse wave velocity (PWVao) and the calculation of the arterial age of the residents. In total, 144 randomly selected residents were included in measurements, divided into the two groups according to Ca and Mg contents in drinking water (water hardness). The first group was supplied with soft drinking water (total dissolved solids (TDS): 200–300 mg·L^−1^, Ca: 20–25 mg·L^−1^, Mg: 5–10 mg·L^−1^). The second group of residents was supplied with harder drinking water (TDS: 500–600 mg·L^−1^, Ca: 80–90 mg·L^−1^, Mg: 25–30 mg·L^−1^). Differences in arterial stiffness between the two groups of respondents were documented. Higher arterial stiffness (low flexibility of arteries) was determined for a group of residents supplied with soft drinking water. This was reflected in higher PWVao levels, higher number of pathological cases (PWVao > 10 m·s^−1^), and arterial age of respondents compared to their actual age. The “absolute” difference between the arterial and actual age between the two evaluated groups of residents (soft vs. harder water) was nearly 5 years on average. The higher arterial stiffness and age of residents that consumed soft drinking water indicate the health significance of lower contents of Ca and Mg in drinking water as an environmental risk factor of cardiovascular diseases. Measuring arterial stiffness of residents in the areas supplied with soft drinking water can be used as a non-invasive approach in the prevention of cardiovascular risks.

## 1. Introduction

The loss of elasticity of arteries, so-called arterial stiffness, is a significant risk factor for the origin of cardiovascular diseases (CVD). Measurement of arterial stiffness, as a non-invasive method in recent years, has become an important part for the predictive determination of cardiovascular risk in preclinical medicine/diagnostics [1,2]. The main CVD risk factors generally include stress, genetic predisposition, obesity, smoking, unhealthy eating habits, and excessive alcohol consumption, but environmental factors (in particular, quality of drinking water and air) are also important [3]. Among the environmental factors, a very important role is played by the chemical composition of drinking water, not only in terms of elevated contents of potentially toxic elements, e.g., As, Cd, Ni, Pb, Sb, and Ba [4,5,6,7,8,9], but also in terms of sufficient contents of elements essential for the human health, mainly Ca and Mg. These two macro-elements represent essential cations, which are involved in many biological processes (metabolic, enzymatic) in humans. Among other functions (e.g., hematopoiesis, the proper function of the heart, etc.), these two elements are important also for the proper development of vascular system [10].

The history of research on the impact of macro-elements in groundwater/drinking water on the human health in relation to CVD can be dated to the beginning of the 1950s. One of the first authors, dealing with the relationship between soft drinking water (deficient contents of Ca and Mg) and CVD occurrence, was Schroeder [11]. He pointed out the need for sufficient Ca and Mg contents in drinking water for the proper development of the vascular system and formulated the results of his research into the well-known compelling dictum: soft water, hard arteries. Recently, mainly in geochemical literature, there are numerous publications documenting the causal link between the increased incidence/mortality for CVD and low contents of Ca and Mg in the drinking water [12,13,14,15,16,17]. In addition, several studies have also been published on the negative effects of desalinated sea water (very low contents of Ca and Mg) on both oncological diseases and diabetes mellitus in recent years [18]. Generally, it is known that the ideal cation ratio of Ca to Mg in drinking water is within the range of 2–3:1. The majority of authors state that contents of Ca and Mg beneficial to human health (including vascular system) are within a range of 20–80 mg·L^−1^ and 10–50 mg·L^−1^, respectively [19]. Despite existing knowledge, no limits of these two elements, legally defining their optimum concentration range in the drinking water (the minimum necessary and the maximum acceptable levels), have yet been established by the World Health Organization [20]. In Slovakia, under the Government Regulation No. 247/2017 Coll., the recommended contents of Ca and Mg in drinking water are >30 mg·L^−1^ and >10 mg·L^−1^, respectively, and the water hardness between 1.1–5.0 mmol·L^−1^.

**Figure 1:**
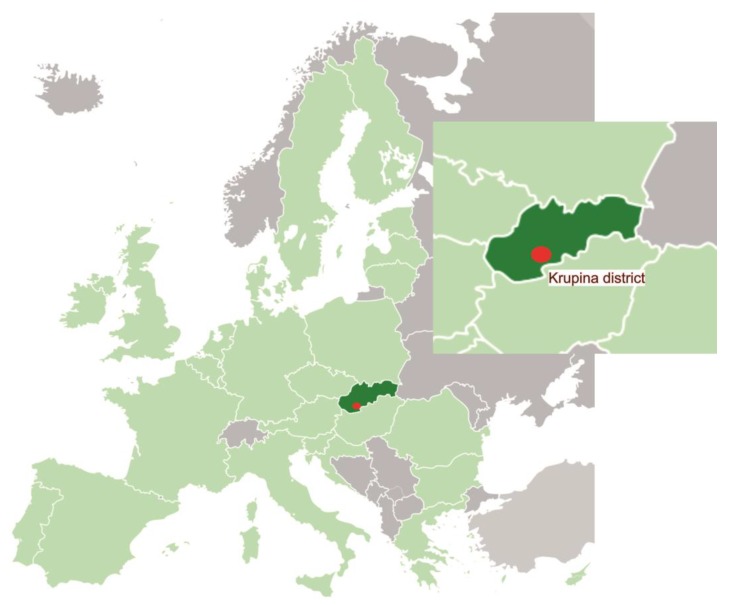
Schematic map showing the location of the study area in the Slovak Republic.

This paper deals with the measurement of arterial stiffness of the residents in the Krupina district, the Slovak Republic (Figure 1), to assess a possible negative impact of low Ca and Mg contents in drinking water on the vascular status of the local population. The study is based on the current knowledge, which indicates a correlation between the unfavourable health status of the population of the Krupina district, mainly regarding the increased mortality for CVDs, and the geological environment of the Neogene volcanic rocks, being reflected mainly in the low Ca and Mg contents in groundwater/drinking waters [15,21,22,23]. The main objective of using arterial stiffness measurements as one of the biomonitoring methods was to identify whether there are differences in arterial age between the two groups of residents in the Krupina district who live in municipalities supplied with drinking water of different hardness.

## 2. Materials and Methods

### 2.1. Area Description

**Figure 2:**
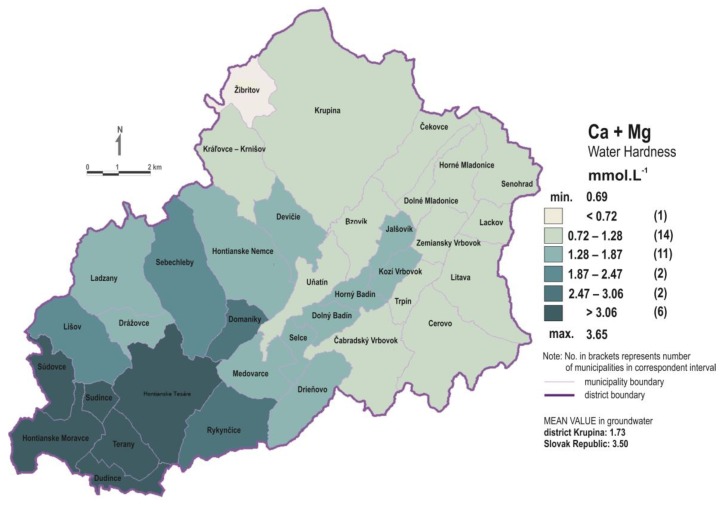
Spatial distribution of groundwater hardness expressed as Ca + Mg (in mmol·L^−1^) within the Krupina district. Note the much higher water hardness in the south-western part of the district.

The Krupina district is a typical rural district of the Slovak Republic. Residents (about 35,000) live mostly in family houses with gardens, where they produce vegetables and fruits for their own consumption. The entire district is geologically built by the Neogene volcanic rocks of the Central Slovakia, mainly the various types of andesites and their pyroclastics. In the southern part of the Krupina district, volcanic rocks gradually descend and are underlain by the Neogene sediments with a dominant carbonate character (sands, gravels, shales, and less limestone). Only low mineralized waters (TDS: 200–300 mg·L^−1^, Ca + Mg < 1.0 mmol·L^−1^) are associated with volcanic rocks due to their low solubility (andesites and their pyroclastics) (Table 1). However, in the southern part of the district, the population is supplied with drinking water from deeper wells, extending into the Neogene bedrock with abundant carbonate sediments (e.g., Dudince, Hontianske Moravce, Hontianske Tesáre, and Terany). These waters are characterized by a much higher mineralization (TDS: 500–600 mg·L^−1^) and hardness (Ca + Mg > 3.0 mmol·L^−1^) (Table 1 and Figure 2). From the point of view of anthropogenic pollution (waters, soils, and sediments), the Krupina district has a very low degree of pollution [24]. In terms of lifestyle and health care levels, no significant differences have been documented in the Krupina district compared to other districts of the Slovak Republic [23].

### 2.2. Selection of Respondents

Respondents included in this study were residents living in the Krupina district, where the majority of municipalities (21) are supplied with soft drinking water and only eight municipalities are supplied with harder drinking water. This study is based on the comparison of the level of arterial stiffness between the two groups of residents from soft and harder drinking water area within the district. The measurement of the arterial stiffness was done in two time phases with a total number of 144 respondents, including 72 respondents from areas with soft drinking water (TDS: 200–300 mg·L^−1^, Ca + Mg < 1.0 mmol·L^−1^) and 72 respondents from regions with harder drinking water (TDS: 500–600 mg·L^−1^, Ca + Mg > 3.0 mmol·L^−1^) (Table 1). The selection of respondents was random, including males and females within the age range of 32–60 years, who live permanently in the Krupina district. The basic selection criteria were as follows: (i) at least five years of residency, (ii) use of drinking water exclusively from a public supply, and (iii) respondents not treated for CVD and other serious chronic diseases, such as diabetes, kidney disease, and chronic inflammation. According to the International Classification of Diseases [25], CVD includes diseases of the circulatory system, diagnosis I.00–I.99. These include hypertension; coronary heart disease (heart attacks); cerebrovascular diseases; and diseases of the arteries, veins, and other non-specified diseases of circulatory system.

**Figure 3:**
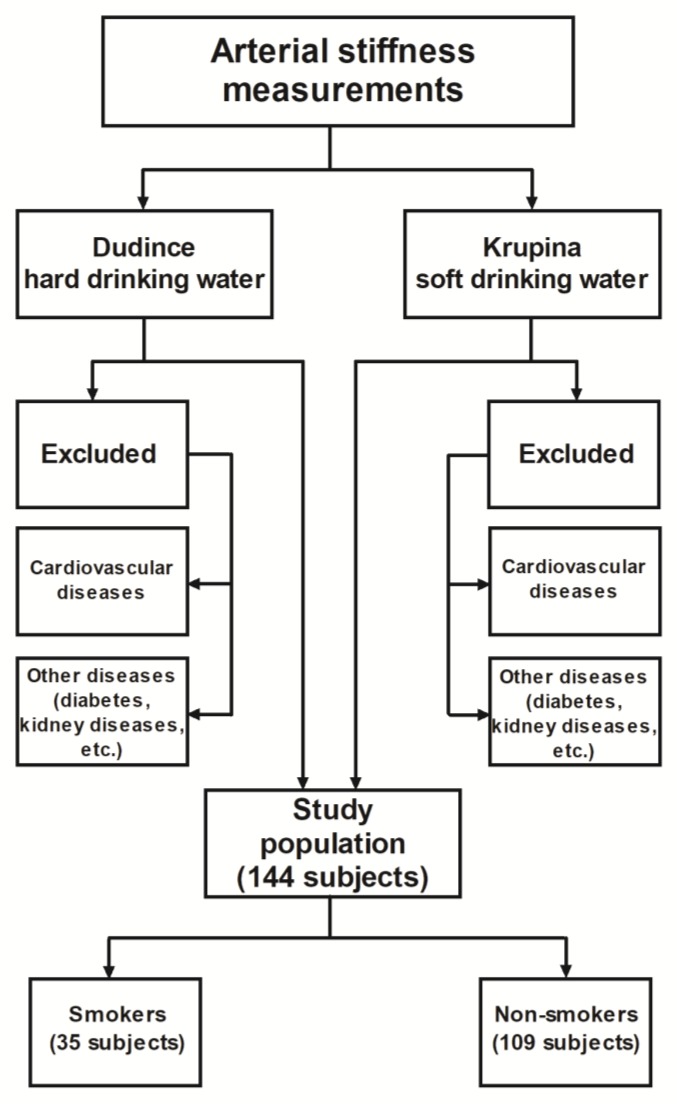
Flowchart showing the selection of respondents included in arterial stiffness measurements.

All participants in the project gave their informed consent for the measurement of arterial stiffness and completed a brief questionnaire on their health status, actual age, height, weight, and smoking habits before taking part in the study. The basic statistics of these characteristics are shown in Table 2 for all individuals participating in this study. The study was performed in accordance with the Declaration of Helsinki, and the protocol was approved by the Ethics Committee of the Regional Public Health Authority of Zvolen (approval no. ZV/KA/2014/1/183). Participation in the research was voluntary, fully anonymous, and participants included in the study were informed that the results of this study would only be used for scientific purposes. The selection of volunteers included in this study is shown in flow chart in Figure 3.

The first phase of the measurement was carried out in May 2014 in two cities of the Krupina district on a sample of 22 selected respondents from the areas supplied with soft water (the Krupina city) and the control group of 22 respondents from the area supplied with harder water (the Dudince city).

The selection of these two cities in the Krupina district was based on the results of the project Life for Krupina [26], which indicated an increased mortality for CVD in the Krupina city, being supplied with drinking water with low Ca and Mg contents, even below the recommended values according the Government Regulation No. 297/2017 Coll. In the Krupina city, a higher relative mortality from diseases of the cardiovascular system has been documented for a period of 1993–2004 with the level of 717.3 deaths per 100,000 inhabitants. In the Dudince city, supplied with harder drinking water (about 3-fold higher contents of Ca and Mg), the level of relative mortality for CVD for this period was significantly lower (484.1 deaths per 100,000 inhabitants). The ReI00–I99 values in the Krupina city greatly exceeded the average of the Slovak Republic in that period with a level of 529.3 deaths per 100,000 inhabitants.

Based on the results obtained in the first phase of arterial stiffness measurement (see below), which showed some differences in arterial age between the two groups of residents, the measurements were repeated with a greater number of respondents. The second phase of measurements was conducted in April 2015 on a sample of 100 respondents, divided in half according to Ca and Mg contents in drinking water (soft, low-mineralized drinking water vs. harder, moderately mineralized drinking water). In the research, both cities (Krupina and Dudince), as well as the surrounding villages, were included. A summary of respondents involved in the measurement of arterial stiffness and of the quality of drinking water in terms of Ca and Mg contents is presented in Table 1.

### 2.3. Measurement of Arterial Stiffness

The term “arterial stiffness” entered into our consciousness only in recent years. The phrase “arterial stiffness” is a general term that refers to the loss of arterial compliance or changes in vessel wall properties, or both [27]. The measurement of arterial stiffness is a simple technique, which was established as a useful non-invasive approach in the health prevention in the past 20 to 25 years [1]. Markers of arterial stiffness, such as increased aortic pulse wave velocity and increased central aortic pressure, are independent predictors of cardiovascular risk [28]. They represent tissue biomarkers of the arteries. These markers have been shown to be better prognosticators than the traditional blood pressure measurement, as well as the biomarkers in the blood stream. In addition, their significant predictive value specifies the risk assessment provided by the traditional risk factors. The arterial stiffness measurement gives insights into the actual pathological processes through the evaluation of the loss of elasticity of the aorta. Over time, the endothelial damage progresses and causes damage to the arterial elasticity, resulting in the loss of elasticity of the vessel wall. In susceptible patients, it leads to premature vascular aging, resulting in the early development of cardiovascular complications. Vascular aging can also be measured directly through the non-invasive method of measuring the arterial stiffness, central pressure, and the wave reflection. In this study, the measurements were performed with an arteriograph developed in Hungary and patented in over 30 countries (Arteriograph, TensioMed Ltd., Budapest, Hungary). The arteriograph can easily measure, without any health risk, such physiological parameters characterizing the state of arteries, which are independent of further known risk factors (age, sex, blood pressure, cholesterol, smoking), and can reliably assess the state of the cardiovascular system and predict the risk of complications in asymptomatic, at first sight “healthy,” patients. These parameters are also confirmed by international guidelines for the diagnosis of the target organ damage [29].

The measurement is carried out with the patient lying down and is similar to the simple blood pressure measurement [30,31]. The measurement can determine more than just the actual systolic and diastolic blood pressure. The cuff attached to the shoulder detects the entire pulse curve, which corresponds to changes in the blood pressure [32]. These curves are subsequently processed by a computer program. The information obtained characterizes the function of small arteries using pressure ratios in the main artery (aorta) in close proximity to the heart and flexibility of the arteries by measuring the aortic pulse wave velocity (PWVao). Based on PWVao values, the so-called arterial age of the individual can be derived. Based on more than 10,000 measurements provided by the company Arteriograph, the “Central European” median PWVao values for different age groups of population were derived, which represents a standard against which the measured results are compared. The relationship between the age and PWVao values for the Central European population is shown in Table 3. With increasing age, PWVao values also increase. Calculation of arterial age represents an integrated software output of the arteriograph. More information about measurements can be obtained online at the website https://youtu.be/JgLklpdzZ0g [33].

In this study, the aorta elasticity was determined through measurements of PWVao and the arterial age of respondents. The measured results were compared with average values established for the Central European population. Pulse wave velocity of 6–10 m∙s^−1^ is considered a standard value [34]. Average arterial age does not correspond to the actual age of an individual but it reflects the status (age) of arteries. The arterial age can be higher or lower than the actual age of the respondent. The more flexible the wall of the arteries, the lower the pulse wave velocity, so the aorta is healthier and its age is lower than the physical, i.e., actual, age of examined person.

### 2.4. Determination of Total Dissolved Solids and Hardness in Drinking Water

Drinking waters analyzed in this study came directly from the water resources that supply residents in the Krupina district. The source of drinking water was exclusively groundwater, which was pumped from wells and captured springs. Water samples were taken at regular intervals in 2014−2015 and water hardness and total dissolved solids were determined in a certified reference laboratory of the State Geological Institute of Dionýz Štúr. The analyses were carried out in accordance with the Slovak drinking water standard (Government Regulation No. 496/2010 Coll., which lays down requirements for water intended for human consumption and quality control of water intended for human consumption). The total dissolved solids (TDS) of drinking water were determined using the gravimetric method on water samples dried at 180 °C [35]. The water hardness was expressed as the sum of the molar concentrations of dissolved Ca and Mg. Calcium and Mg were measured using inductively coupled plasma-optical emission spectrometry (ICP-OES; Liberty 200, Varian, Palo Alto, CA, USA).

### 2.5. Statistical Analysis

The results of the measurements were expressed using average, median, standard deviation, and range (minimum–maximum). An independent sample *t* test and Mann–Whitney test were used to evaluate statistical differences between groups of populations living in the hard water and soft water areas. The *z*-test and the Monte Carlo method were used to test differences in the proportions of males and females, and smokers and non-smokers among populations living in the hard drinking water and soft drinking water areas. Multivariate analysis of variance (MANOVA) was also performed to test whether other factors (sex, age, smoking, and body mass index) affected the measured variables, especially the values of PWVao and the difference between arterial and actual age. Statistical processing of the results was done using XLSTAT software (Addinsoft Inc, Long Island City, NY, USA). Statistical significance was considered to be *p* < 0.05.

## 3. Results

**Figure 4:**
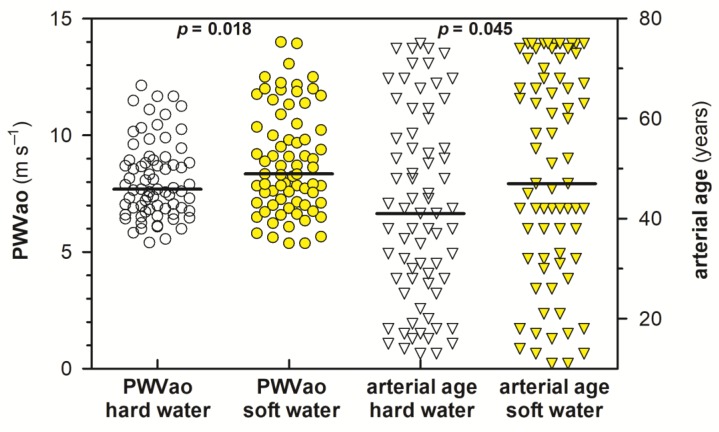
Scatter plot showing aortic pulse wave velocity (PWVao) values and arterial age of residents living in areas with different contents of Ca and Mg in drinking water. Horizontal continuous lines are the median values. White and yellow circles represent the individual PWVao values of the residents from hard and soft drinking water areas, respectively, while the white and yellow inverted triangles are the calculated arterial ages of the residents from hard and soft drinking water areas, respectively.

**Figure 5:**
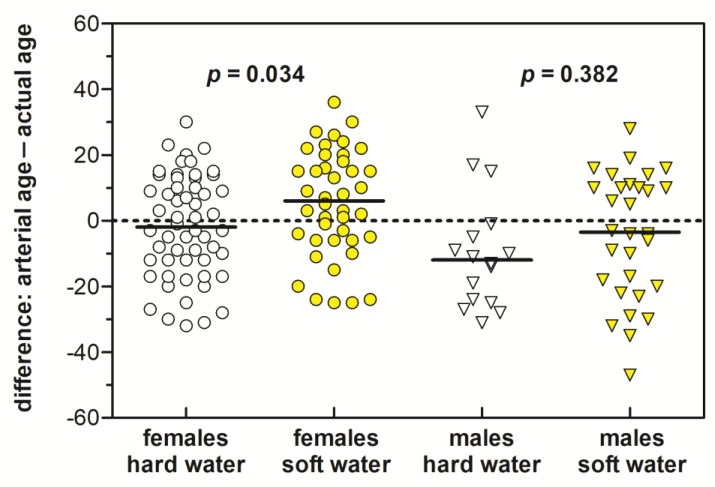
Differences between the arterial and actual age of residents living in areas with soft and hard drinking water. Horizontal continuous lines are the median values.

The results of arterial stiffness measurements for the first, second, and both phases are summarized in Table 4. Differences in PWVao values and arterial age of respondents as a function of water hardness are presented in the form of scatter plots in Figure 4. In addition, Figure 5 illustrates graphically the difference between the arterial and actual age of females and males for both measurement phases. As can be seen from Table 4 and Figure 4, the average PWVao values and arterial age were significantly higher in areas with low contents of Ca and Mg in drinking water than in areas with higher Ca and Mg contents in water (*p* < 0.05). The difference between the arterial and actual age also indicated a somewhat more unfavorable health status (higher median in Figure 5) of respondents using soft drinking water; however, the difference was significantly higher only in females (*p* < 0.05). The greater the difference, the worse the arterial health.

To investigate the effects of other possible factors on arterial health, multivariate analysis of variance was used. The results of this analysis indicated that risk factors, such as smoking and BMI, did not affect PWVao values, arterial age, and the difference between arterial and actual age in any of the two areas studied (Table 5). The influence of sex was not confirmed, and only age affected these variables. However, the average actual age of the population from the hard water area did not differ significantly from the age of the population from the soft water area (Table 2 and Table 4).

## 4. Discussion

The results of arterial stiffness measurements documented differences in arterial age and PWVao values between the two groups of residents who live in the Krupina district in cities/villages supplied with drinking water of different hardness (i.e., different contents of Ca and Mg). These differences have been documented in both phases of the research. The more favorable artery status was observed in the areas supplied with hard drinking water compared to areas supplied with soft water. The difference was mainly due to the lower occurrence of pathological cases with abnormally high levels of PWVao (PWVao > 10 m·s^−1^), smaller differences between the arterial and actual age of individuals, and more favorable values of arterial age compared to the Central European average.

In the first phase of measurements, out of 22 respondents from the area supplied with harder drinking water, 10 of them had an arterial age worse than their actual age and 2 had a pathological result (PWVao >10 m·s^−1^). Two residents had an average result and the arterial age of 10 residents was better than their actual age. Compared to the average values for the Central European population, the respondents had an arterial age better (lower) by approximately 3.60 years on average. On the other hand, of the 22 respondents living in the area supplied with soft drinking water, the arterial age was worse than the actual age for 11 residents, and 5 had a pathological result (PWVao > 10 m·s^−1^) (Table 4). Five residents had average results and 6 had a better arterial age than their actual age. Respondents had an average arterial age that was worse by 1.91 years than the average in Central Europe. Based on the comparison of the results of the first phase of arterial stiffness measurements in both groups of residents, it could be documented that the “absolute” difference between the actual and arterial age of the population supplied with soft drinking water (the Krupina city) was approximately 5.5 years higher than that for respondents who are supplied with harder drinking water (the Dudince city).

In the second phase of measurements, out of 50 residents living in the area supplied with harder drinking water, arterial ages of 19 residents were worse than their respective actual age, including 9 residents with a pathological result (PWVao > 10 m·s^−1^). Four residents had average results and better arterial age was documented in 27 residents. On average, the residents living in areas with harder drinking water had an arterial age approximately 3.40 years better than the Central European average. However, in areas with soft drinking water, the arterial age was worse than the actual age in 29 residents, while 16 of them exhibited PWVao values higher than 10 m·s^−1^ (Table 4). One resident had an average result and 20 residents presented a better arterial age than their respective actual age. The residents had on average an arterial age that was 0.96 years worse than the Central European average. The values of PWVao were also generally higher compared to the PWVao values obtained from measurements of residents from the control group (areas with the harder drinking water). The “absolute” difference between the arterial and actual age of the population supplied with soft drinking water was 4.32 years higher than that of respondents from the harder drinking water area (Table 4). These findings confirmed the results from the first phase of measurements. This was also reflected by higher PWVao levels, increased number of pathological conditions, and consequently, by a higher arterial age of residents compared to their actual age.

The results, including all 144 respondents who participated in the measurement of arterial stiffness, indicated the potential impact of soft drinking water on the impaired status of arteries of the population. This was particularly due to a greater number of extreme PWVao values, and subsequently, a significantly higher arterial age of residents living in soft drinking water area (Figure 4 and Table 4), and a higher deviation from the values established for the Central European population. In addition, as can be seen from Table 4, it was found that the difference between the arterial and actual age of residents supplied with soft drinking water was on average (including both the first and second phases of measurements) 4.67 years higher compared to the residents supplied with hard drinking water. However, when the median values of this difference were compared, the “absolute” difference between hard water area and soft water area was even higher, up to 9 years.

Small differences between the arterial and actual age were also observed according to sex, i.e., between males and females (Figure 5), but we consider these results only as being indicative due to the different numbers of males and females in both evaluated groups of residents and their variable age structure. In the case of both males as well as females, a better status of arteries in inhabitants from areas supplied with hard drinking water was observed. Statistical comparison of the results between the two groups using the unpaired *t* test showed that the difference between the arterial and actual age in females was significantly higher in areas with soft drinking water than in those with hard drinking water (*p* < 0.05; Figure 5). However, no statistically significant difference in this parameter was confirmed in males (Figure 5), which might be related to the small number of males included in the study. However, the data presented in Table 4 and Figure 4 showed a significantly higher prevalence (*p* < 0.05) of higher arterial age and elevated PWVao values in residents from the soft drinking water area, while the physical age of both groups of residents was almost identical (*p* > 0.05; Table 2 and Table 4).

These results can hardly be confronted with other works because, to our best knowledge, there is no literature documenting the relationship between arterial stiffness and water hardness. However, it should be noted that medical studies clearly show the protective role of Mg and Ca for arteries [36,37,38]. Magnesium deficiency has been shown to have a detrimental effect on arterial elasticity [39,40], and it is well known that the state of arteries has a direct relationship to the development of cardio-vascular diseases [27]. In a previous study on the impact of groundwater chemical composition on the relative mortality of Slovakia’s population for cardiovascular diseases, Rapant et al. [15] demonstrated a significant positive association of elevated Ca and Mg contents in groundwater with a decrease of relative mortality. Several works from other countries around the world have shown a positive, i.e., protective, significance of water hardness and optimal Ca and Mg contents against cardiovascular disease mortality [14,41,42,43]. In particular, it is possible to state the work of Yousefi et al. [43] who observed the association between drinking water hardness and the risk of hypertension in the population of Poldasht County in Iran. They have shown that the prevalence of hypertension is significantly higher in areas with low water hardness than in those with higher water hardness. It was further observed in Iran that the number of cardiovascular diseases per 1000 inhabitants was lower in areas with drinking water containing more than ≈70 mg·L^−1^ Ca and ~30 mg·L^−1^ Mg [44]. These results are consistent with findings from Slovakia, where the lowest mortality rate for cardiovascular diseases is associated with areas that have drinking water with Ca content above ≈80 mg·L^−1^ and Mg above ≈40 mg·L^−1^ [15]. However, it should be noted that there are also studies that have shown no relationship between water hardness and cardiovascular diseases [45,46].

## 5. Conclusions

This study showed higher arterial stiffness (lower flexibility of arteries) and higher arterial age in residents who are supplied with soft drinking water (with Ca and Mg deficiency) compared to the group of residents with a hard drinking water supply. The “absolute” difference between the arterial and actual age of two groups of respondents (soft vs. harder water) was almost 5.5 years and 4.3 years in the first and second phase of measurements, respectively. Averaging all measured data, the difference was higher by 4.7 years in the group of residents supplied with soft drinking water. If the median values are used for comparison, the difference reached up to 9 years. It should be noted that all residents included in this study live in neighboring cities/villages with the same health determinants, such as lifestyle, socio-economic factors, health care, genetic factors, eating habits, etc., and the same age structure.

The results of the two-phased arterial stiffness measurements support the assumption of a potentially negative impact of the unfavourable geological environment, reflected in the low mineralization and hardness of drinking water, as a significant environmental factor on the health status of the population living in the Krupina district. It could be concluded that low contents of Ca and Mg in drinking water and low water hardness (Ca + Mg) negatively affected the status of arteries of the residents from the Krupina district through higher arterial stiffness and higher arterial age. We find it necessary to verify our results in other countries around the world.

Based on the results, it is recommended that arterial stiffness measurements are incorporated into the indicators of cardiovascular risk/mortality for cardiovascular diseases that should be used in areas where the population is supplied with soft drinking water with low Ca and Mg levels. If our findings are confirmed in other countries, it will be another reason to include Ca and Mg among regulated elements in the WHO drinking water standard.

## Figures and Tables

**Table 1:** Summary of participants involved in the measurements of arterial stiffness and chemical composition of drinking water in terms of Ca and Mg contents.

Municipality	Number of Males	Number of Females	All	Ca (mg·L^−1^)	Mg (mg·L^−1^)	Ca + Mg (mmol·L^−1^)
The First Phase of Measurements						
Krupina ^1^	6	16	22	23.6	9.2	0.97
Dudince ^2^	9	13	22	96.8	21.3	3.29
The Second Phase of Measurements						
Krupina ^1^	9	16	25	23.6	9.2	0.97
Horné Mladonice ^1^	6	2	8	22.9	7.2	0.87
Litava ^1^	5	4	9	21.5	7.4	0.84
Senohrad ^1^	4	4	8	22.9	5.1	0.78
Dudince ^2^	2	23	25	96.8	21.3	3.29
Hontianske Moravce ^2^	2	11	13	86.5	26.8	3.34
Hontianske Tesáre ^2^	3	9	12	88.2	28.0	3.35

^1^ area with soft drinking water; ^2^ area with harder drinking water.

**Table 2:** Summary of basic characteristics of participants involved in the study.

Statistical Parameter	Total	Dudince—Hard Drinking Water	Krupina—Soft Drinking Water	*p*-Value
Actual Age				
Average ± SD ^1^	46.3 ± 7.66	45.3 ± 7.58	47.4 ± 7.65	0.105 ^2^
Median	45.0	44.0	48.0	
Range	32–60	32–59	35–60	
Body Mass Index (BMI)				
Average ± SD	25.5 ± 2.51	25.2 ± 2.29	25.8 ± 2.70	0.191 ^2^
Median	25.4	25.3	25.9	
Range	18.4–32.3	19.5–30.6	18.4–32.3	
Sex				
Male	46 (31.9%)	16 (22.2%)	30 (41.7%)	0.018 (0.015) ^3^
Female	98 (68.1%)	56 (77.8%)	42 (58.3%)	0.018 (0.015)
Smoking				
Smokers	35 (24.3%)	20 (27.8%)	15 (20.8%)	0.436 (0.287) ^3^
Non-smokers	109 (75.7%)	52 (72.2%)	57 (79.2%)	0.436 (0.287)

^1^ standard deviation; ^2^ based on unpaired *t* test; ^3^ based on *z*-test and Monte Carlo simulations in parentheses.

**Table 3:** Median PWVao values and age of the Central European population according to Reference [33].

Age (years)	10	15	20	25	30	35	40	45	50	55	60
PWVao (m·s^−1^)	5.35	6.00	6.60	6.80	7.00	7.30	7.70	8.30	8.60	8.80	9.00

**Table 4:** Common statistical parameters of the measured characteristics of respondents in the 1^st^, 2^nd^ and both research phases.

Statistical Parameter	Dudince—Hard Drinking Water				Krupina—Soft Drinking Water			
	Actual Age (years)	Arterial Age (years)	Difference ^1^	PWVao ^2^ (m·s^−1^)	Actual Age (years)	Arterial Age (years)	Difference	PWVao (m·s^−1^)
The First Phase (*n* = 44) ^3^								
Range	32–59	13–71	−31 to +30	5.41–11.1	35–59	13–75	−25 to +36	5.63–12.5
Average	44.6	41.0	−3.55	7.97	45.4	48.3	+1.91	8.70
Median	44.0	43.5	−4.00	7.79	45.0	46.5	0.00	8.29
SD^4^	8.03	18.3	16.5	1.45	8.53	19.4	14.2	2.02
PWVao^5^ > 10 m·s^−1^				2				5
*p*-value^6^	0.482	0.211	0.246	0.186				
The Second Phase (*n* = 100)								
Range	35–59	13–75	−32 to +33	5.56–12.1	36–60	11–75	−47 to +30	5.38–14.0
Average	45.6	42.3	−3.36	8.14	47.8	48.8	+0.96	8.95
Median	44.0	39.5	−5.00	7.67	48.0	47.0	+7.00	8.35
SD	7.44	19.4	16.5	1.74	7.28	22.0	19.5	2.33
PWVao > 10 m·s^−1^				9				16
*p*-value	0.173	0.119	0.293	0.050				
All Measurements (*n* = 144)								
Range	32–59	13–75	−32 to +33	5.41–12.1	35–60	11–75	−47 to +36	5.38–14.0
Average	45.3	41.9	−3.42	8.09	47.4	48.6	+1.25	8.87
Median	44.0	41.0	−5.00	7.69	48.0	47.0	+4.00	8.35
SD	7.58	18.9	16.4	1.65	7.65	21.1	18.0	2.23
PWVao > 10 m·s^−1^				11				21
*p*-value	0.105	0.045	0.106	0.018				

^1^ the difference is calculated as arterial age – actual age; positive values mean that arterial age is higher than actual age (i.e., worse status of arteries) and vice versa; ^2^ aortic pulse wave velocity; ^3^ number of respondents; ^4^ standard deviation; ^5^ number of respondents with a pathological outcome; ^6^ based on *t* test.

**Table 5:** Results of multivariate variance analysis (MANOVA; Lawley–Hotelling trace test).

Statistics	Dudince—Hard Drinking Water				Krupina—Soft Drinking Water			
	Age	Sex	BMI ^1^	Smoking	Age	Sex	BMI	Smoking
Lambda	0.767	0.052	0.169	0.011	0.365	0.085	0.043	0.015
F (observed)	8.435	1.179	1.859	0.254	4.013	1.923	0.475	0.338
DF1 ^2^	6	3	6	3	6	3	6	3
DF2 ^2^	132	68	132	68	132	68	132	68
F (critical)	2.168	2.740	2.168	2.740	2.168	2.740	2.168	2.740
*p*-value	<0.0001	0.324	0.093	0.858	0.001	0.134	0.826	0.798

^1^ body mass index; ^2^ degrees of freedom.
